# 12,15-Dimethyl-8-oxa­tetra­cyclo­[8.8.0.0^2,7^.0^11,16^]octa­deca-1(18),2,4,6,11(16),12,14-heptaen-10-ol

**DOI:** 10.1107/S2414314620003156

**Published:** 2020-03-10

**Authors:** Alan J. Lough, Samuel Koh, William Tam

**Affiliations:** a Department of Chemistry, University of Toronto, Toronto, Ontario, M5S 3H6, Canada; b Department of Chemistry, University of Guelph, Guelph, Ontario, N1G 2W1, Canada; University of Aberdeen, Scotland

**Keywords:** crystal structure, ring-opening, regioselectivity, hydrogen bonds

## Abstract

In the title compound, C_19_H_18_O_2_, the pyran ring is in a half-chair conformation. The fused ring system comprising the benzene and cyclo­hexene rings is essentially planar (r.m.s. deviation = 0.053 Å) and forms a dihedral angle of 27.95 (6)° with the other benzene ring. In the crystal, O—H⋯O hydrogen bonds connect the mol­ecules into chains propagating along [001].

## Abstract 2

In the title compound, he pyran ring is in a half-chair conformation and the fused ring system comprising the benzene and cyclo­hexene rings is essentially planar and forms a dihedral angle of 27.95 (6)° with the other benzene ring. In the crystal, O—H⋯O hydrogen bonds connect the mol­ecules into chains propagating along [001].

## Graphical abstract

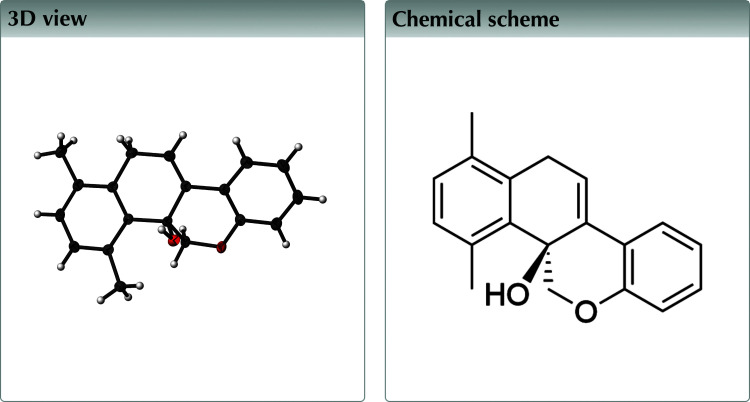

## Structure description

**Figure 1:**
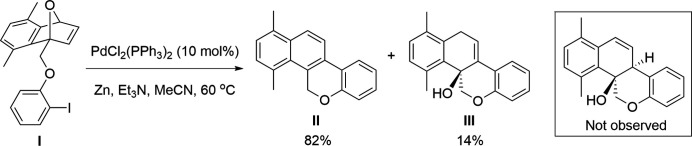
The reaction scheme

The ring-opening reaction of oxabenzonorbornadiene (OBD) has been well studied by many groups including our own (Lautens *et al.*, 2003 ▸; Rayabarapu & Cheng, 2007 ▸; Boutin *et al.*, 2019 ▸; Hill *et al.*, 2019 ▸; Hill & Tam, 2019 ▸). Building on the work of Cheng (Duan & Cheng, 1995 ▸), our group has also demonstrated the palladium-catalysed regioselective ring-opening of C_1_-substituted OBDs using aryl iodides (Raheem *et al.*, 2014 ▸). However, to the best of our knowledge, intra­molecular modes of this reactivity have been left unexplored. Currently, the only known intra­molecular transformation of OBD was reported by the Lautens group (Loh *et al.*, 2016 ▸) with a similar transformation recently reported by our group on cyclo­propanated OBD (Wicks *et al.*, 2019 ▸). Based on this, we set out to investigate palladium-catalysed intra­molecular ring-openings of OBD with C_1_-tethered aryl halides. The reaction of C_1_-substituted OBD **I** (see Fig. 1 ▸) in the presence of PdCl_2_(PPh_3_)_2_, Zn, Et_3_N, and MeCN afforded an expected dehydrated product **II** in 82% yield, as well as an unexpected and yet unreported hydrated product **III** in 14% yield. The structure of the alcohol-containing fused tetra­cycle **III** was confirmed by single-crystal X-ray analysis.

**Figure 2:**
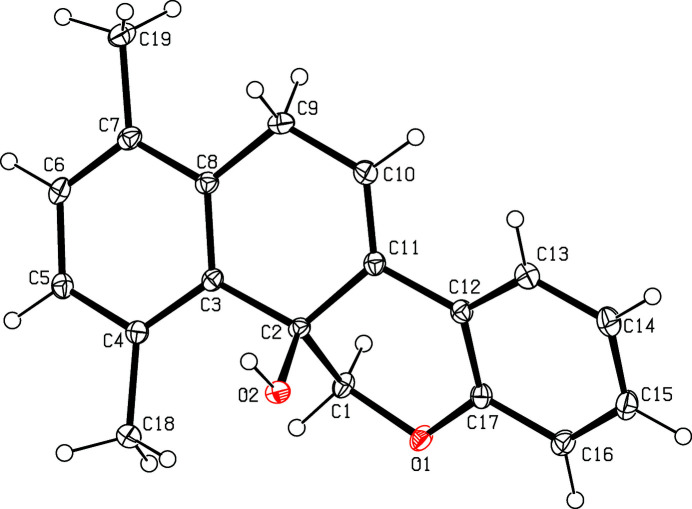
The mol­ecular structure of the title compound with displacement ellipsoids drawn at the 30% probability level.

**Figure 3:**
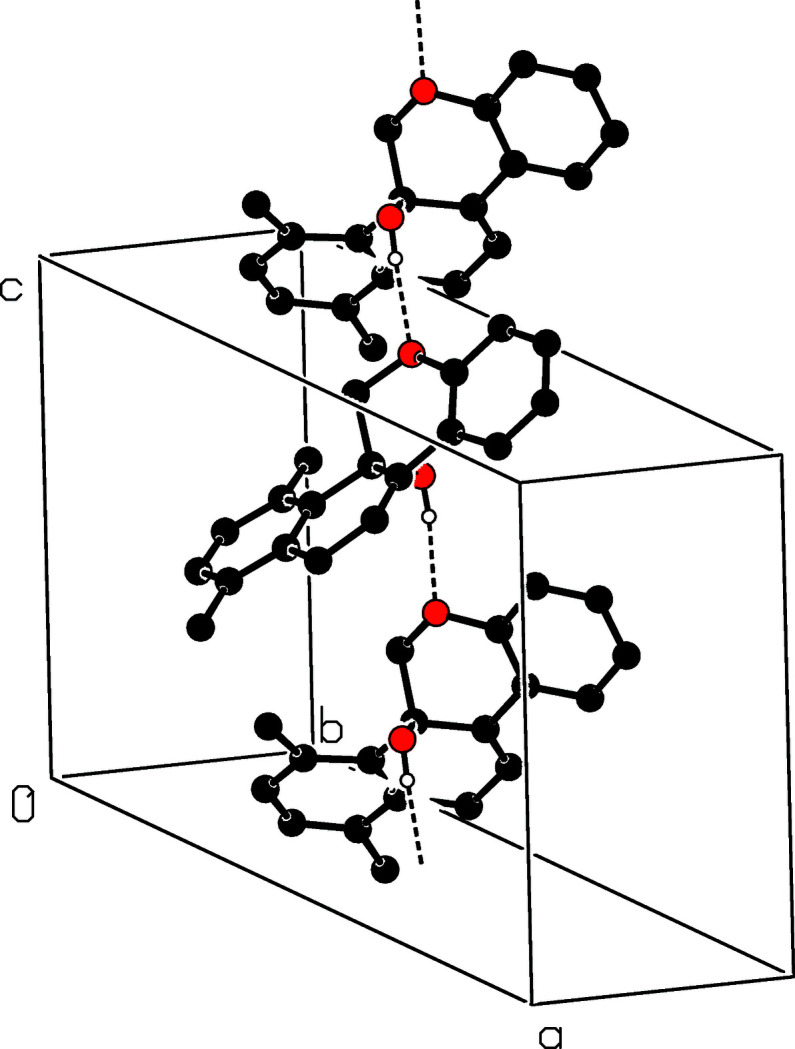
Part of the crystal structure with O—H⋯O hydrogen bonds shown as dashed lines.

The mol­ecular structure of the title compound is shown in Fig. 2 ▸. The pyran ring (O1/C1/C2/C11/C12/C17) is in a half-chair conformation with atoms C1 and C2 deviating from the mean-plane of the other four atoms by −0.197 (2) and 0.556 (1) Å, respectively. The fused ring system comprising the benzene (C3–C8) and cyclo­hexene (C2/C3/C8–C11) rings is essentially planar (r.m.s. deviation = 0.053 Å) and forms a dihedral angle of 27.95 (6)° with the other benzene ring (C12–C17). In the arbitrarily chosen asymmetric unit, atom C2 has an *S* configuration but crystal symmetry generates a racemic mixture. In the crystal, O—H⋯O hydrogen bonds (Table 1 ▸) connect the mol­ecules into chains propagating along [001] (Fig. 3 ▸).

## Synthesis and crystallization

To a 2 dram vial was added oxabenzonorbornadiene **I** (Fig. 1 ▸) (67.8 mg, 0.168 mmol), then purged with argon before importing into a glove box under an inert argon atmosphere. The vial was loaded sequentially with Zn (123.3 mg, 1.89 mmol, 11.2 eq.), MeCN (1.5 ml), Et_3_N (0.09 ml, 0.669 mmol, 0.25 eq.) and PdCl_2_(PPh_3_)_2_ (12.5 mg, 0.0178 mmol, 10.6 mol%), then exported and stirred at 333 K for 1 day. The mixture was cooled to room temperature and stirred in air for 10 minutes before removing the solvent under reduced pressure. The crude mixture was then purified by flash column chromatography using gradient elution (EtOAc:hexa­nes 1:9 to EtOAc:hexa­nes 1:4) to obtain the ring-opened product **II** (35.8 mg, 82%) as a white solid and **III** (6.6 mg, 14%) as a white solid. The product **III** was subsequently crystallized from methyl­ene chloride solution by slow evaporation to give product **III** as colourless crystals with orange specks.

## Refinement

Crystal data, data collection and structure refinement details are summarized in Table 2 ▸.

## Supplementary Material

Crystal structure: contains datablock(s) I. DOI: 10.1107/S2414314620003156/hb4341sup1.cif

Structure factors: contains datablock(s) I. DOI: 10.1107/S2414314620003156/hb4341Isup2.hkl

Supporting information file. DOI: 10.1107/S2414314620003156/hb4341Isup3.cml

CCDC reference: 1988571

Additional supporting information:  crystallographic information; 3D view; checkCIF report

## Figures and Tables

**Table 1:** Hydrogen-bond geometry (Å, °)

*D*—H⋯*A*	*D*—H	H⋯*A*	*D*⋯*A*	*D*—H⋯*A*
O2—H2*O*⋯O1^i^	0.86 (2)	2.03 (2)	2.8805 (14)	171.0 (19)

Symmetry code: (i) 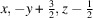 .

**Table 2:** Experimental details

Crystal data	
Chemical formula	C_19_H_18_O_2_
*M*_r_	278.33
Crystal system, space group	Monoclinic, *P*2_1_/*c*
Temperature (K)	150
*a*, *b*, *c* (Å)	12.2712 (7), 11.2934 (6), 10.8984 (7)
β (°)	112.565 (2)
*V* (Å^3^)	1394.71 (14)
*Z*	4
Radiation type	Mo *K*α
μ (mm^−1^)	0.09
Crystal size (mm)	0.25 × 0.19 × 0.11

Data collection	
Diffractometer	Bruker Kappa *APEX* DUO CCD
Absorption correction	Multi-scan (*SADABS*; Krause *et al.*, 2015 ▸)
*T*_min_, *T*_max_	0.703, 0.746
No. of measured, independent and observed [*I* > 2σ(*I*)] reflections	22859, 3212, 2404
*R*_int_	0.040
(sin θ/λ)_max_ (Å^−1^)	0.650

Refinement	
*R*[*F* ^2^ > 2σ(*F* ^2^)], *wR*(*F* ^2^), *S*	0.040, 0.110, 1.04
No. of reflections	3212
No. of parameters	196
H-atom treatment	H atoms treated by a mixture of independent and constrained refinement
Δρ_max_, Δρ_min_ (e Å^−3^)	0.31, −0.22

Computer programs: *APEX3* (Bruker, 2018 ▸), *SAINT* (Bruker, 2018 ▸), *SHELXT2014* (Sheldrick, 2015*a* ▸), *SHELXL2018* (Sheldrick, 2015*b* ▸), *PLATON* (Spek, 2020 ▸), *publCIF* (Westrip, 2010 ▸).

## full crystallographic data

### Crystal data

**Table:**

C_19_H_18_O_2_	*F*(000) = 592
*M**_r_* = 278.33	*D*_x_ = 1.326 Mg m^−^^3^
Monoclinic, *P*2_1_/*c*	Mo *K*α radiation, λ = 0.71073 Å
*a* = 12.2712 (7) Å	Cell parameters from 6674 reflections
*b* = 11.2934 (6) Å	θ = 2.6–27.5°
*c* = 10.8984 (7) Å	µ = 0.09 mm^−^^1^
β = 112.565 (2)°	*T* = 150 K
*V* = 1394.71 (14) Å^3^	Shard, colourless
*Z* = 4	0.25 × 0.19 × 0.11 mm

### Data collection

**Table:**

Bruker Kappa APEX DUO CCD diffractometer	2404 reflections with *I* > 2σ(*I*)
Radiation source: sealed tube with Bruker Triumph monochromator	*R*_int_ = 0.040
φ and ω scans	θ_max_ = 27.5°, θ_min_ = 1.8°
Absorption correction: multi-scan (SADABS; Krause *et al.*, 2015)	*h* = −15→15
*T*_min_ = 0.703, *T*_max_ = 0.746	*k* = −14→12
22859 measured reflections	*l* = −14→14
3212 independent reflections	

### Refinement

**Table:**

Refinement on *F*^2^	Primary atom site location: dual
Least-squares matrix: full	Hydrogen site location: mixed
*R*[*F*^2^ > 2σ(*F*^2^)] = 0.040	H atoms treated by a mixture of independent and constrained refinement
*wR*(*F*^2^) = 0.110	*w* = 1/[σ^2^(*F*_o_^2^) + (0.0478*P*)^2^ + 0.6316*P*] where *P* = (*F*_o_^2^ + 2*F*_c_^2^)/3
*S* = 1.03	(Δ/σ)_max_ < 0.001
3212 reflections	Δρ_max_ = 0.31 e Å^−^^3^
196 parameters	Δρ_min_ = −0.21 e Å^−^^3^
0 restraints	

### Special details

**Table:**

Geometry. All esds (except the esd in the dihedral angle between two l.s. planes) are estimated using the full covariance matrix. The cell esds are taken into account individually in the estimation of esds in distances, angles and torsion angles; correlations between esds in cell parameters are only used when they are defined by crystal symmetry. An approximate (isotropic) treatment of cell esds is used for estimating esds involving l.s. planes.

### Fractional atomic coordinates and isotropic or equivalent isotropic displacement parameters (Å^2^)

**Table:**

	*x*	*y*	*z*	*U*_iso_*/*U*_eq_	
O1	0.38362 (9)	0.71483 (9)	0.94095 (10)	0.0227 (2)	
O2	0.35317 (9)	0.79851 (9)	0.68983 (10)	0.0195 (2)	
H2O	0.3639 (17)	0.8028 (17)	0.616 (2)	0.041 (6)*	
C1	0.28530 (12)	0.67697 (13)	0.82436 (13)	0.0194 (3)	
H1A	0.216856	0.729079	0.811291	0.023*	
H1B	0.263028	0.595336	0.838378	0.023*	
C2	0.31342 (12)	0.67990 (12)	0.69883 (13)	0.0165 (3)	
C3	0.20305 (12)	0.64875 (12)	0.57662 (13)	0.0168 (3)	
C4	0.10115 (12)	0.72098 (12)	0.54010 (14)	0.0183 (3)	
C5	−0.00046 (13)	0.68454 (13)	0.43491 (14)	0.0207 (3)	
H5A	−0.070186	0.730804	0.411431	0.025*	
C6	−0.00220 (13)	0.58267 (13)	0.36392 (14)	0.0217 (3)	
H6A	−0.073619	0.558560	0.294864	0.026*	
C7	0.09851 (13)	0.51547 (13)	0.39205 (14)	0.0204 (3)	
C8	0.20197 (12)	0.54850 (12)	0.49950 (14)	0.0185 (3)	
C9	0.31027 (13)	0.47357 (14)	0.52591 (15)	0.0266 (3)	
H9A	0.289357	0.389843	0.532701	0.032*	
H9B	0.333308	0.479937	0.448491	0.032*	
C10	0.41426 (13)	0.50446 (14)	0.64767 (15)	0.0230 (3)	
H10A	0.482918	0.456604	0.670766	0.028*	
C11	0.41632 (12)	0.59543 (12)	0.72595 (13)	0.0182 (3)	
C12	0.51602 (12)	0.61900 (12)	0.85341 (14)	0.0188 (3)	
C13	0.63234 (13)	0.58519 (13)	0.87853 (15)	0.0235 (3)	
H13A	0.649773	0.547125	0.810468	0.028*	
C14	0.72254 (13)	0.60601 (14)	1.00035 (16)	0.0266 (3)	
H14A	0.800938	0.582302	1.015531	0.032*	
C15	0.69775 (13)	0.66181 (14)	1.10041 (16)	0.0258 (3)	
H15A	0.759337	0.675389	1.184465	0.031*	
C16	0.58418 (13)	0.69762 (13)	1.07837 (15)	0.0231 (3)	
H16A	0.567555	0.736584	1.146479	0.028*	
C17	0.49430 (12)	0.67609 (13)	0.95542 (14)	0.0192 (3)	
C18	0.09345 (14)	0.83818 (14)	0.60380 (16)	0.0255 (3)	
H18A	0.172468	0.872790	0.644921	0.038*	
H18B	0.060772	0.825342	0.671871	0.038*	
H18C	0.042111	0.892264	0.535837	0.038*	
C19	0.09555 (14)	0.40895 (14)	0.30771 (15)	0.0271 (3)	
H19A	0.154520	0.418330	0.268138	0.041*	
H19B	0.016948	0.401618	0.237010	0.041*	
H19C	0.113242	0.337581	0.363080	0.041*	

### Atomic displacement parameters (Å^2^)

**Table:**

	*U*^11^	*U*^22^	*U*^33^	*U*^12^	*U*^13^	*U*^23^
O1	0.0194 (5)	0.0335 (6)	0.0143 (5)	0.0017 (4)	0.0056 (4)	−0.0035 (4)
O2	0.0245 (5)	0.0175 (5)	0.0182 (5)	−0.0023 (4)	0.0100 (4)	−0.0002 (4)
C1	0.0176 (7)	0.0263 (8)	0.0140 (7)	−0.0015 (6)	0.0059 (5)	−0.0003 (5)
C2	0.0197 (7)	0.0162 (7)	0.0141 (6)	−0.0010 (5)	0.0071 (5)	−0.0006 (5)
C3	0.0191 (7)	0.0186 (7)	0.0136 (6)	−0.0012 (5)	0.0073 (5)	0.0013 (5)
C4	0.0223 (7)	0.0181 (7)	0.0162 (7)	0.0000 (6)	0.0091 (6)	0.0019 (5)
C5	0.0203 (7)	0.0231 (8)	0.0182 (7)	0.0023 (6)	0.0068 (6)	0.0035 (6)
C6	0.0220 (7)	0.0255 (8)	0.0146 (7)	−0.0036 (6)	0.0036 (6)	0.0013 (6)
C7	0.0264 (7)	0.0195 (7)	0.0155 (7)	−0.0032 (6)	0.0083 (6)	0.0001 (5)
C8	0.0223 (7)	0.0183 (7)	0.0160 (7)	−0.0010 (5)	0.0085 (6)	0.0000 (5)
C9	0.0279 (8)	0.0252 (8)	0.0239 (8)	0.0039 (6)	0.0068 (6)	−0.0078 (6)
C10	0.0225 (7)	0.0247 (8)	0.0214 (7)	0.0041 (6)	0.0079 (6)	−0.0007 (6)
C11	0.0192 (7)	0.0202 (7)	0.0159 (7)	0.0005 (5)	0.0076 (5)	0.0024 (5)
C12	0.0206 (7)	0.0179 (7)	0.0176 (7)	−0.0003 (6)	0.0071 (6)	0.0021 (5)
C13	0.0231 (7)	0.0234 (8)	0.0243 (8)	0.0018 (6)	0.0094 (6)	0.0022 (6)
C14	0.0194 (7)	0.0278 (8)	0.0298 (8)	0.0025 (6)	0.0065 (6)	0.0041 (7)
C15	0.0229 (8)	0.0256 (8)	0.0221 (8)	−0.0028 (6)	0.0010 (6)	0.0033 (6)
C16	0.0263 (8)	0.0242 (8)	0.0177 (7)	−0.0018 (6)	0.0073 (6)	0.0003 (6)
C17	0.0189 (7)	0.0203 (7)	0.0180 (7)	−0.0001 (5)	0.0064 (6)	0.0032 (6)
C18	0.0250 (8)	0.0242 (8)	0.0245 (8)	0.0053 (6)	0.0063 (6)	−0.0030 (6)
C19	0.0320 (9)	0.0247 (8)	0.0215 (8)	−0.0029 (6)	0.0070 (6)	−0.0061 (6)

### Geometric parameters (Å, º)

**Table:**

O1—C17	1.3771 (17)	C9—H9A	0.9900
O1—C1	1.4410 (17)	C9—H9B	0.9900
O2—C2	1.4417 (17)	C10—C11	1.329 (2)
O2—H2O	0.86 (2)	C10—H10A	0.9500
C1—C2	1.5343 (18)	C11—C12	1.4809 (19)
C1—H1A	0.9900	C12—C17	1.397 (2)
C1—H1B	0.9900	C12—C13	1.399 (2)
C2—C11	1.5184 (19)	C13—C14	1.384 (2)
C2—C3	1.5322 (19)	C13—H13A	0.9500
C3—C8	1.4071 (19)	C14—C15	1.390 (2)
C3—C4	1.4162 (19)	C14—H14A	0.9500
C4—C5	1.393 (2)	C15—C16	1.381 (2)
C4—C18	1.514 (2)	C15—H15A	0.9500
C5—C6	1.382 (2)	C16—C17	1.392 (2)
C5—H5A	0.9500	C16—H16A	0.9500
C6—C7	1.381 (2)	C18—H18A	0.9800
C6—H6A	0.9500	C18—H18B	0.9800
C7—C8	1.408 (2)	C18—H18C	0.9800
C7—C19	1.506 (2)	C19—H19A	0.9800
C8—C9	1.507 (2)	C19—H19B	0.9800
C9—C10	1.487 (2)	C19—H19C	0.9800
			
C17—O1—C1	117.52 (11)	H9A—C9—H9B	107.5
C2—O2—H2O	107.0 (13)	C11—C10—C9	123.53 (14)
O1—C1—C2	112.41 (11)	C11—C10—H10A	118.2
O1—C1—H1A	109.1	C9—C10—H10A	118.2
C2—C1—H1A	109.1	C10—C11—C12	123.11 (13)
O1—C1—H1B	109.1	C10—C11—C2	123.44 (13)
C2—C1—H1B	109.1	C12—C11—C2	113.31 (12)
H1A—C1—H1B	107.9	C17—C12—C13	117.53 (13)
O2—C2—C11	108.61 (11)	C17—C12—C11	119.23 (12)
O2—C2—C3	111.32 (11)	C13—C12—C11	123.23 (13)
C11—C2—C3	114.62 (11)	C14—C13—C12	121.44 (14)
O2—C2—C1	106.17 (11)	C14—C13—H13A	119.3
C11—C2—C1	105.53 (11)	C12—C13—H13A	119.3
C3—C2—C1	110.11 (11)	C13—C14—C15	119.62 (14)
C8—C3—C4	119.39 (13)	C13—C14—H14A	120.2
C8—C3—C2	120.44 (12)	C15—C14—H14A	120.2
C4—C3—C2	120.18 (12)	C16—C15—C14	120.41 (14)
C5—C4—C3	118.42 (13)	C16—C15—H15A	119.8
C5—C4—C18	116.22 (13)	C14—C15—H15A	119.8
C3—C4—C18	125.34 (13)	C15—C16—C17	119.37 (14)
C6—C5—C4	121.55 (13)	C15—C16—H16A	120.3
C6—C5—H5A	119.2	C17—C16—H16A	120.3
C4—C5—H5A	119.2	O1—C17—C16	115.93 (13)
C7—C6—C5	120.87 (13)	O1—C17—C12	122.45 (12)
C7—C6—H6A	119.6	C16—C17—C12	121.62 (13)
C5—C6—H6A	119.6	C4—C18—H18A	109.5
C6—C7—C8	118.93 (13)	C4—C18—H18B	109.5
C6—C7—C19	119.58 (13)	H18A—C18—H18B	109.5
C8—C7—C19	121.49 (13)	C4—C18—H18C	109.5
C3—C8—C7	120.59 (13)	H18A—C18—H18C	109.5
C3—C8—C9	122.15 (13)	H18B—C18—H18C	109.5
C7—C8—C9	117.25 (13)	C7—C19—H19A	109.5
C10—C9—C8	115.51 (12)	C7—C19—H19B	109.5
C10—C9—H9A	108.4	H19A—C19—H19B	109.5
C8—C9—H9A	108.4	C7—C19—H19C	109.5
C10—C9—H9B	108.4	H19A—C19—H19C	109.5
C8—C9—H9B	108.4	H19B—C19—H19C	109.5
			
C17—O1—C1—C2	−41.25 (17)	C7—C8—C9—C10	−174.41 (13)
O1—C1—C2—O2	−54.75 (14)	C8—C9—C10—C11	−3.5 (2)
O1—C1—C2—C11	60.43 (14)	C9—C10—C11—C12	174.19 (14)
O1—C1—C2—C3	−175.37 (11)	C9—C10—C11—C2	−1.2 (2)
O2—C2—C3—C8	123.95 (13)	O2—C2—C11—C10	−122.26 (14)
C11—C2—C3—C8	0.19 (18)	C3—C2—C11—C10	2.93 (19)
C1—C2—C3—C8	−118.58 (14)	C1—C2—C11—C10	124.26 (15)
O2—C2—C3—C4	−55.88 (16)	O2—C2—C11—C12	61.99 (14)
C11—C2—C3—C4	−179.64 (11)	C3—C2—C11—C12	−172.82 (11)
C1—C2—C3—C4	61.59 (16)	C1—C2—C11—C12	−51.50 (14)
C8—C3—C4—C5	5.28 (19)	C10—C11—C12—C17	−150.86 (15)
C2—C3—C4—C5	−174.89 (12)	C2—C11—C12—C17	24.91 (18)
C8—C3—C4—C18	−172.91 (13)	C10—C11—C12—C13	28.1 (2)
C2—C3—C4—C18	6.9 (2)	C2—C11—C12—C13	−156.10 (13)
C3—C4—C5—C6	−2.2 (2)	C17—C12—C13—C14	0.8 (2)
C18—C4—C5—C6	176.20 (13)	C11—C12—C13—C14	−178.20 (14)
C4—C5—C6—C7	−2.3 (2)	C12—C13—C14—C15	−0.1 (2)
C5—C6—C7—C8	3.6 (2)	C13—C14—C15—C16	−0.7 (2)
C5—C6—C7—C19	−176.41 (13)	C14—C15—C16—C17	0.8 (2)
C4—C3—C8—C7	−4.1 (2)	C1—O1—C17—C16	−169.75 (12)
C2—C3—C8—C7	176.07 (12)	C1—O1—C17—C12	10.5 (2)
C4—C3—C8—C9	174.87 (13)	C15—C16—C17—O1	−179.75 (13)
C2—C3—C8—C9	−5.0 (2)	C15—C16—C17—C12	0.0 (2)
C6—C7—C8—C3	−0.3 (2)	C13—C12—C17—O1	178.97 (13)
C19—C7—C8—C3	179.64 (13)	C11—C12—C17—O1	−2.0 (2)
C6—C7—C8—C9	−179.36 (13)	C13—C12—C17—C16	−0.8 (2)
C19—C7—C8—C9	0.6 (2)	C11—C12—C17—C16	178.27 (13)
C3—C8—C9—C10	6.6 (2)		

### Hydrogen-bond geometry (Å, º)

**Table:**

*D*—H···*A*	*D*—H	H···*A*	*D*···*A*	*D*—H···*A*
O2—H2*O*···O1^i^	0.86 (2)	2.03 (2)	2.8805 (14)	171.0 (19)

Symmetry code: (i) *x*, −*y*+3/2, *z*−1/2.
